# Photoperiod effects in a freshwater community: Amphibian larvae develop faster and zooplankton abundance increases under an early‐season photoperiod

**DOI:** 10.1002/ece3.10400

**Published:** 2023-08-08

**Authors:** Troy C. Neptune, Michael F. Benard

**Affiliations:** ^1^ Department of Biology Case Western Reserve University Cleveland Ohio USA

**Keywords:** anuran, climate, development, global change, phenology

## Abstract

Organisms that shift their phenologies in response to global warming will experience novel photic environments, as photoperiod (daylength) continues to follow the same annual cycle. How different organisms respond to novel photoperiods could result in phenological mismatches and altered interspecific interactions. We conducted an outdoor mesocosm experiment exposing green frog (*Rana clamitans*) larvae, gray treefrog (*Hyla versicolor*) larvae, phytoplankton, periphyton, and zooplankton to a three‐month shift in photoperiod: an early‐season photoperiod (simulating April) and a late‐season photoperiod (simulating July). We manipulated photoperiod by covering and uncovering tanks with clear or light‐blocking lids to mimic realistic changes in daylength. We assessed amphibian life history traits and measured phytoplankton, periphyton, and zooplankton abundances. Green frog larvae and gray treefrog metamorphs were more developed under the early‐season photoperiod. Gray treefrog total length was also reduced, but photoperiod did not affect green frog total length. Although phytoplankton and periphyton abundances were not affected by photoperiod, copepod nauplii were in greater abundance under the early‐season photoperiod. Overall, this simplified aquatic community did not exhibit significant changes to structure when exposed to a three‐month shift in photoperiod. Temperate amphibians that breed earlier in the year may develop faster, which may have long‐term costs to post‐metamorphic growth and performance. Asynchronous shifts in zooplankton abundances in response to altered photoperiods could subsequently affect freshwater community structure. While photoperiod has been shown to individually affect freshwater organisms, our study using replicated outdoor wetland communities shows that the comprehensive effects of photoperiod may be less important than other cues such as temperature and precipitation.

## Graphical abstract

We conducted a field‐based, mesocosm experiment exposing a simplified freshwater community (amphibians, phytoplankton, periphyton, and zooplankton) to a three‐month shift in photoperiod. Our results demonstrate that both amphibians developed faster and copepod reproduction increased under the early‐season photoperiod. Remarkably, our results suggest that this temperate freshwater community did not exhibit significant changes in structure despite being exposed to photoperiods representing two distinct seasons (spring and summer).

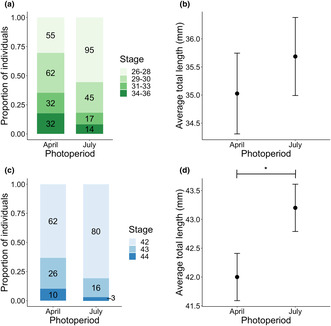

## 1 INTRODUCTION

As global change alters patterns of temperature and precipitation, photoperiod continues to follow the same annual cycle at any given location. Organisms use changes in photoperiod, or daylength, as cues to initiate life history events such as diapause, migration, reproduction, and development (Bradshaw & Holzapfel, 2007). However, many taxa are changing their phenology in response to rising temperatures (Cohen et al., 2018; Ge et al., 2015; Ovaskainen et al., 2013). Organisms that shift their phenology with temperature will subsequently experience novel photic environments. Novel photic conditions (e.g. different photoperiods) may disrupt the development, behavior, and physiology of individual organisms, potentially resulting in changes to interspecific interactions (Donnelly et al., 2011; Visser & Both, 2005; Walker et al., 2019). Freshwater communities are excellent systems in which to study photoperiod because amphibians, phytoplankton, and zooplankton have been shown to individually respond to photoperiod, but how these organisms respond to photoperiod in the context of a community is uncertain.

Amphibians are one such group where phenological shifts are especially apparent, yet the effects of photoperiod are largely unknown. Amphibians are breeding earlier in the year as climate change progresses (Gibbs & Breisch, 2001; Parmesan, 2007; Todd et al., 2011; While & Uller, 2014). These phenological shifts consequently expose amphibian larvae to novel photic conditions throughout ontogeny. Few studies, however, directly test how photoperiod affects amphibian life history traits (Burraco et al., 2020; Kukita et al., 2015; Laurila et al., 2001), with some data suggesting that certain amphibian larvae develop faster under relatively short photoperiods in some (Kukita et al., 2015), but not all studies (Burraco et al., 2020; Laurila et al., 2001). Furthermore, studies like Kukita et al. (2015) and Burraco et al. (2020) use fixed photoperiods under laboratory conditions. Fixed photoperiods, such as 18 h light:6 h dark, do not fluctuate like natural photoperiods and lack biological realism. Experiments that simulate changing photoperiods will provide a better test for how photoperiod signals seasonality and how that signal impacts interacting organisms in freshwater communities.

While photoperiod may directly affect amphibians, photoperiod may also indirectly affect developing amphibian larvae through changes in the abundances of phytoplankton, periphyton, and zooplankton. Phytoplankton and zooplankton phenologies have advanced with climate change in both freshwater (Adrian et al., 2006) and marine systems (Edwards & Richardson, 2004). Specifically in freshwater systems, peak abundances of diatoms have greatly advanced, while patterns of peak abundances of zooplankton are mixed (Adrian et al., 2006; Winder & Schindler, 2004). With these shifts in phenology, plankton will also experience novel photoperiods. Changes in the availability and timing of these food sources could consequently affect amphibian growth responses as larval amphibians primarily consume phytoplankton and periphyton (but see Arribas et al., 2015; Schiesari et al., 2009). Therefore, photoperiod may indirectly influence amphibian growth and development by directly affecting the abundances of phytoplankton, periphyton, and zooplankton.

Although some plankton phenologies are changing, research on the effects of photoperiod on phytoplankton, periphyton, and zooplankton is limited. Phytoplankton growth (change in biomass) often increases with longer photoperiods, presumably through longer total light exposure (Seyfabadi et al., 2011; Shatwell et al., 2012; Tang & Vincent, 2000; Theus et al., 2022; but see Litchman, 2000). Additionally, longer photoperiods can increase the overall phytoplankton species richness in a community (Celewicz & Gołdyn, 2021). Regarding periphyton, photoperiod does not seem to affect biomass (Chaumet et al., 2020; Laderriere et al., 2022). In comparison to phytoplankton and periphyton, zooplankton appear to exhibit more diverse responses to variation in photoperiod. Some studies find increased cladoceran and copepod abundances under long photoperiods and high temperatures, but responses to photoperiod are mixed when assessing rotifer abundances (Dupuis & Hann, 2009; Jones & Gilbert, 2016). Similar to the research on amphibians, many studies of plankton use fixed photoperiods (Celewicz & Gołdyn, 2021; Dupuis & Hann, 2009; Jones & Gilbert, 2016). While one can infer how changing photoperiods (i.e. changes in total light exposure) may influence traits such as phytoplankton growth, there are no studies that examine how realistic changes in photoperiod simultaneously affect phytoplankton, periphyton, and zooplankton under field conditions.

In this study, we used an outdoor mesocosm experiment to investigate whether an early‐season photoperiod modified a freshwater community relative to a late‐season photoperiod to better understand the complex outcomes of phenological shifts under continued warming. We manipulated photoperiod during the larval development of two anurans from different families, the green frog (Ranidae: *Rana clamitans*) and the eastern gray treefrog (Hylidae: *Hyla versicolor*). Green frogs predominantly overwinter as larvae in northern latitudes but can metamorphose within the same season if eggs are laid early in the year (Berven et al., 1979; Martof, 1956). We, therefore, predicted green frogs to develop faster under the early‐season photoperiod to metamorphose within the same season, rather than overwintering as larvae. Conversely, gray treefrogs have shorter larval periods and can only metamorphose in the same season in which they are laid. We predicted gray treefrogs in the late‐season photoperiod (i.e. perceiving a time constraint) would develop faster to metamorphose before the end of the season (Johansson & Rowe, 1999; Kukita et al., 2015). Earlier metamorphosis is associated with increased post‐metamorphic survivorship and growth (Altwegg & Reyer, 2003). However, models predict organisms that develop faster under time constraints will do so at the cost of a smaller size at metamorphosis (Abrams et al., 1996; Rowe & Ludwig, 1991). We thus predicted anuran larvae that develop faster to metamorphose at a smaller size. We also assessed how phytoplankton, periphyton, and zooplankton differed in abundance between the two photoperiods. We predicted that phytoplankton abundance would increase under the longer (late‐season) photoperiod due to longer light exposure (Seyfabadi et al., 2011; Shatwell et al., 2012; Tang & Vincent, 2000; Theus et al., 2022), but we had no *a priori* predictions for periphyton and zooplankton.

## 2 MATERIALS AND METHODS

### 2.1 Study design

We conducted an outdoor mesocosm experiment in 1000 L cattle tanks at Case Western Reserve University's Squire Valleevue and Valley Ridge Farm (hereafter CWRU Farm), Hunting Valley, Ohio, USA (41°29′34″ N, 81°25′26″ W). Each mesocosm was randomly assigned to receive either green frogs or gray treefrogs and one of two photoperiod treatments. Each species–photoperiod combination was replicated eight times for a total of 32 mesocosms. Mesocosms were arranged in four blocks of eight mesocosms.

We added approximately 700 L of strained pond water to each mesocosm on June 7, 2021, which excluded predatory insects but retained phytoplankton, periphyton, and zooplankton. We added each of the following to every mesocosm: 11.4 L of leaf litter as a substrate and as supplemental nutrients on June 8, 2021, one ramshorn snail (*Planorbella trivolvis*) on June 15, 2021, two additional snails on June 22, 2021, and 20 g of alfalfa rabbit food as an initial food source on June 30, 2021. On June 29, 2021, we floated HOBO® pendant temperature data loggers (±0.53°C) 10 cm below the water surface in 16 mesocosms. We only had 16 data loggers available for this experiment, and thus we were unable to place data loggers in each tank. Therefore, we evenly divided the data loggers between the two photoperiod treatments to record temperature every 15 min with four data loggers per block (one data logger malfunctioned; thus, we used recordings from 15 mesocosms). We also added floating platforms wrapped with mesh for metamorphosing frogs to climb and rest upon on June 29, 2021. We covered each tank with 60% shade cloth (Ecologic Technologies, Inc.) to prevent predation or colonization. These shade lids remained on the mesocosms at all times, including during the photoperiod manipulations.

Our two photoperiod treatments simulated the natural photoperiod that larvae would have experienced if their parents bred at different times of the year: early‐season (simulating breeding in late March and larval development beginning 1 April) or late‐season (simulating breeding in late June and larval development beginning 1 July). We have found green frog eggs from May until August at our field site (C. T. Neptune, M. F. Benard, unpublished data). Gray treefrogs primarily breed in mid‐May to mid‐June in Ohio, but males have been known to call in early‐April (Pfingsten et al., 2013). The early‐season photoperiod used in our experiment would likely represent a photoperiod not currently experienced by either species when developing as larvae at this particular location in northeastern Ohio. But both species are widespread across latitude and thus experience variation in photoperiod during larval development. To manipulate photoperiod, we covered and uncovered mesocosms with light‐blocking (i.e. early‐season) or clear (i.e. late‐season) plastic lids to mimic realistic daylength changes over time, representing different sunset times (Table A1). We used clear plastic sheeting to make the clear lids and black plastic sheeting for the black lids (each measuring 152‐micron in thickness). We covered the mesocosms each evening with the plastic lids and then removed them after sunset when darkness had completely fallen. The plastic lids were on the mesocosms during the time period corresponding to the difference in daylength between the two photoperiods (Table A1). Daylengths for the study site were obtained from *timeanddate* (https://www.timeanddate.com).

We added 25 hatchlings of either species to each mesocosm. We collected three green frog egg masses from the same pond at the CWRU Farm on two dates: one gathered on June 25, 2021, and two gathered on June 27, 2021. We added a total of 25 green frog hatchlings to 16 mesocosms on June 30, 2021: nine hatchlings from one clutch and eight hatchlings from each of the other two clutches. The photoperiod manipulation started upon adding the green frog larvae, marking day 1 of the experiment. For the gray treefrogs, we collected one egg clutch on June 27, 2021, and a second egg clutch on July 6, 2021. We added 13 hatchlings from the first clutch to the other 16 mesocosms on July 1, 2021, and 12 hatchlings from the second clutch on July 9, 2021 (hereafter Clutch 1 and Clutch 2, consecutively). Gray treefrogs naturally have a prolonged breeding season lasting over 2 months. We wanted to include more than one family, but egg availability was limited, which is why we used this staggered approach. However, the addition of the second gray treefrog clutch allowed us to assess differences in photoperiod responses at two points in development: at metamorphosis (Clutch 1) and late larval development (Clutch 2).

### 2.2 Data collection

Beginning on July 23, 2021, we inspected each mesocosm daily for metamorphosing frogs, identified by the presence of the emergence of at least one front limb (Gosner, 1960). All metamorphs were euthanized in buffered 0.02% tricaine methanesulfonate (Finquel brand MS‐222) and preserved in 70% ethanol. We recorded the date of preservation as our measure of age at metamorphosis (date preserved minus egg collection date). This process was repeated daily until all metamorphs were collected. We collected gray treefrogs from the first clutch as they metamorphosed from days 24 to 33 (99 metamorphs from the early‐season treatment and 100 metamorphs from the late‐season treatment). We also collected 11 metamorphs from the second clutch on days 34 to 36 and euthanized and preserved them as previously described.

**FIGURE A1:**
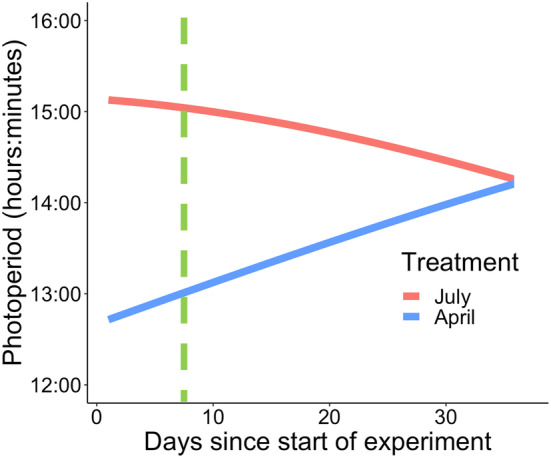
During the experiment, daylength increased daily in the early‐season (April) photoperiod and decreased daily in the late‐season (July) photoperiod. At the beginning of the experiment, the early‐season (April) photoperiod was approximately 2 h and 24 min shorter than the late‐season (July) photoperiod. The green dashed line represents the day the Clutch 2 gray treefrog larvae were added to the mesocosms.

Because the natural photoperiod had decreased below the amount of light needed for the simulated early‐season photoperiod, we had to terminate the experiment (Figure A1). So, on days 37 and 38 (final 2 days of experiment), we gathered the remaining 179 gray treefrog and 352 green frog larvae by carefully sifting leaf litter and draining water from the mesocosms. We then euthanized and preserved these larvae as previously described. We recorded all morphological metrics of the preserved individuals in the lab using digital calipers (e.g. total length, mass, snout‐vent length, head width, tibiofibula length, and total leg length), except mass for which we used a digital scale (OHAUS Pioneer™ 0.01 g precision). We also assigned development stages to all individuals (Gosner, 1960).

To determine if photoperiod also affected phytoplankton and zooplankton abundance, we collected four 1 L water samples, one at each cardinal direction, 40 cm from the mesocosm edge and approximately 10 cm below the water surface from each mesocosm on days 10, 23, and 36 of the experiment. As a relative measure of phytoplankton abundance, we measured chlorophyll *a* fluorescence of four 1 mL subsamples from each 1 L sample using an Aquafluor® fluorometer (Turner Designs), following the methods of Stoler et al. (2017). The four 1 L water samples were then sieved through a 64‐micron filter to collect zooplankton. Zooplankton samples were preserved in 50 mL of 95% ethanol. We then counted cladocerans, copepods, and their nauplii, and rotifers from five 1 mL subsamples using a Sedgwick‐Rafter counting cell for each mesocosm. We also calculated a Shannon‐Weiner Diversity index on zooplankton for each subsample. To measure relative periphyton growth, we suspended one white hexagonal tile (23.4 cm^2^) approximately 25 cm below the water surface in each mesocosm on day 21 of the experiment (the tile was added late in the experiment due to logistical constraints). At the end of the experiment (days 37–38), we scraped all periphyton from the tile surface into 50 mL tubes filled with 15 mL of distilled water and recorded fluorescence with the fluorometer.

### 2.3 Data analysis

For most analyses, we fit linear mixed‐effects models using the “lmer” function from the “lme4” package (Bates et al., 2015). To ensure that our photoperiod manipulation did not affect mesocosm temperature, we first modeled mesocosm temperature (temperatures recorded during each manipulation) with photoperiod treatment and block as predictors and date‐time as a random factor. We also tested for block effects on temperature across the entire day (date‐time as a random factor). While there were no effects of block on temperature, we opted to test for effects of block on all outcomes and dropped block from the model if it was not significant. We then tested for effects of photoperiod on amphibian traits, phytoplankton abundance, and zooplankton abundance using linear mixed‐effects models which included mesocosm as a random factor (Tables A2 and A3).

For the periphyton abundance model, we used a linear model with photoperiod and frog species as predictors without random effects, as we had only one measurement for each mesocosm. For all phytoplankton abundance, zooplankton abundance, and zooplankton Shannon–Weiner Diversity index models, we also included frog species as a predictor and separately fit models for each sampling date. To model the count data, we fit zooplankton abundance models with a Poisson distribution and a log link function using the “glmmTMB” function from the “glmmTMB” package (Brooks et al., 2017).

Depending on the outcome variable, we fit linear mixed‐effects models with different fixed effects for all amphibian measurements (Table A2). For all development stage models across both species, we used cumulative link mixed models from the “ordinal” package (Christensen, 2019) to fit models with the development stage as a categorial outcome. Development stages for each model were collapsed to ensure appropriate model fitting. To fit the development stage model for green frogs, we collapsed stages into four categories: 26–28, 29–30, 31–33, and 34–36 (Gosner, 1960). To fit the gray treefrog larvae model (Clutch 2), we also collapsed stages into four categories: 26–30, 31–35, 36–39, and 41–43 (Gosner, 1960).

For the green frog and gray treefrog larval models with mass and total length as outcomes, we controlled for stage by including the development stage as a covariate (continuous). We excluded 11 stage 42 and 43 gray treefrog metamorphs from analyses on mass and total length for Clutch 2 larvae to maintain linearity, as body size decreases as tadpoles approach and complete metamorphosis (Smith‐Gill & Berven, 1979). All of the excluded tadpoles were from the same mesocosm and early‐season photoperiod. Gray treefrog metamorph (Clutch 1) development stage ranged from Gosner 42 to 46 (Gosner, 1960). We excluded two metamorphs from our analyses (one stage 45 individual from the late‐season photoperiod and one stage 46 individual from the early‐season photoperiod), as these animals were missed during an earlier daily collection; thus, the development model only included metamorph stages 42, 43, and 44. For all gray treefrog metamorph models, we included age at metamorphosis and development stage as covariates, except for when the development stage and age at metamorphosis were outcomes in the models. We also included snout‐vent length as a covariate when head width, tibiofibula length, and total leg length were outcomes, as they scale with body size (Berner, 2011).

We obtained *p*‐values using likelihood‐ratio tests for all linear mixed effects models using “ANOVA.” We also tested for significance of predictors for the periphyton fluorescence (tile) model using “ANOVA” with an accompanying *F*‐test. Interactions are included when significant. All analyses were conducted in RStudio Build 554 using R version 4.2.1 (R Core Team, 2022). Data manipulation and visualization were conducted using the “tidyverse” package (Wickham et al., 2019).

## 3 RESULTS

### 3.1 Photoperiod and temperature

At the beginning of the experiment, the early‐season (April) photoperiod was about 2 h and 24 min shorter than the late‐season (July) photoperiod (Figure A1). During the experiment, the early‐season photoperiod increased each day, while the late‐season photoperiod decreased (Figure A1). Neither photoperiod treatments nor block affected average daily temperature (photoperiod: *χ* ^2^ = 0.00, *p* = .988; block: *χ* ^2^ = 1.70, *p* = .636) or average temperature during the photoperiod manipulation (photoperiod: *χ* ^2^ = 0.00, *p* = .987; block: *χ* ^2^ = 1.16, *p* = .762). The average daily temperature across all mesocosms was 24.18°C ± 0.01 SE, the average daily maximum was 26.40°C ± 0.08 SE, the average daily minimum was 22.31°C ± 0.07 SE, and the average daily coefficient of variation was 5.59 ± 0.11 SE. There was no difference in the total number of metamorphs recovered between the photoperiod treatments (early‐season: 12.25 ± 0.37 SE per mesocosm; late season: 12.50 ± 0.28 SE per mesocosm; *F*_1,14_ = 0.30, *p* = .590).

### 3.2 Anuran life history traits

**FIGURE 1:**
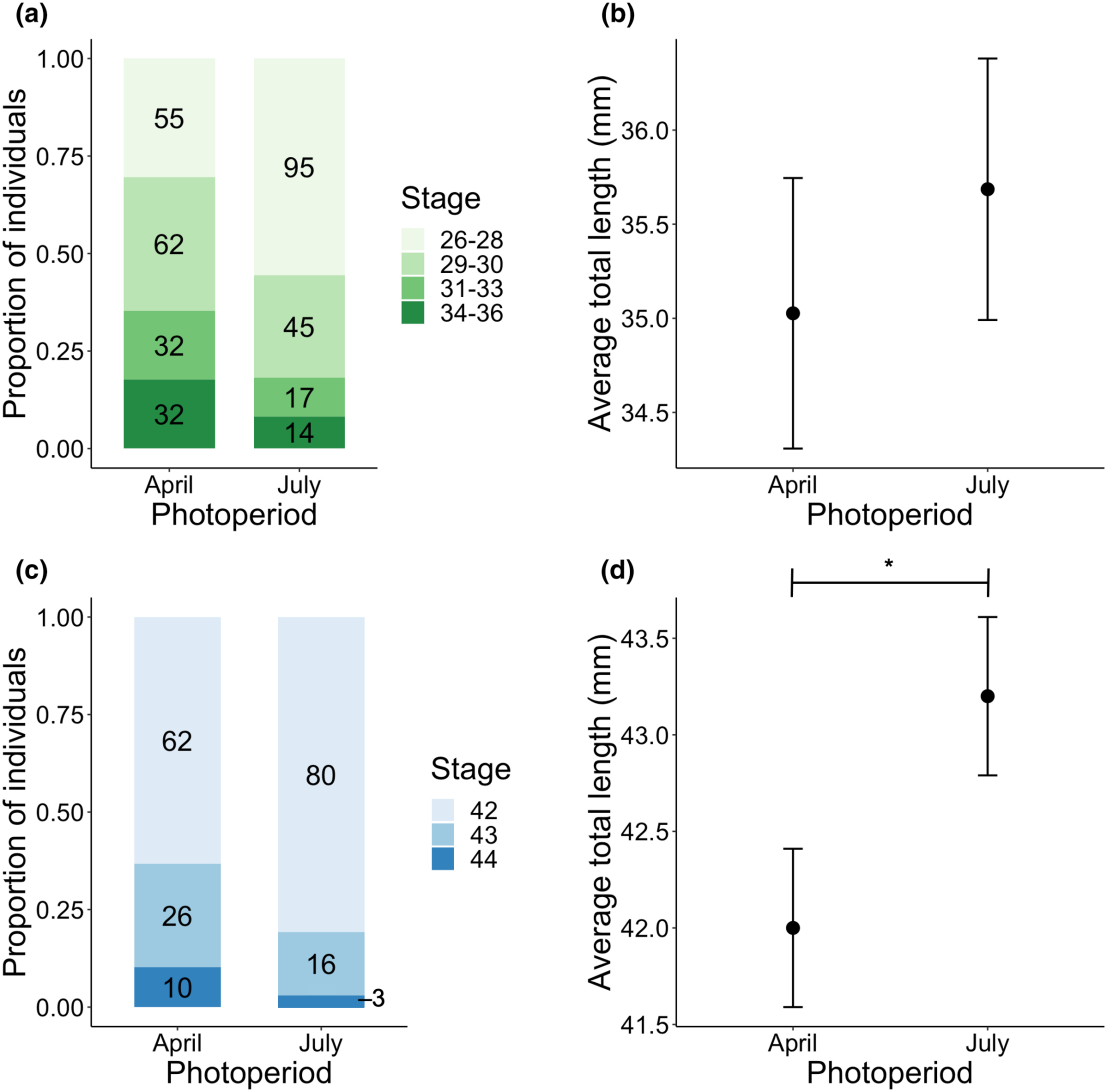
(a) Green frog larvae were marginally more developed under the early‐season (April) photoperiod (*χ* ^2^ = 3.26, *p* = .056). Development stages were collapsed into four categories: 26–28, 29–30, 31–33, and 34–36. Numbers in black indicate the total number of individuals in that category. (b) Green frog average total length did not vary with photoperiod (*χ* ^2^ = 0.02, *p* = .884). Estimated marginal means and standard error bars are plotted. (c) Gray treefrogs significantly accelerated development during metamorphosis (Gosner stages 42–44) under the early‐season (April) photoperiod (*χ* ^2^ = 7.52, *p* = .006). Numbers in black inside the color blocks indicate the total number of individuals in that stage. (d) Average total length (snout to tail tip) of gray treefrog metamorphs was significantly shorter in the early‐season (April) photoperiod while controlling for development stage (*χ* ^2^ = 3.90, *p* = .048). Estimated marginal means and standard error bars are plotted.

We found a marginally significant effect of photoperiod on green frog larval development, with the early‐season (April) photoperiod larvae developing faster (*χ* ^2^ = 3.65, *p* = .056; Figure 1a). However, photoperiod did not affect green frog larval mass (*χ* ^2^= 0.62, *p* = .431) or total length (*χ* ^2^ = 0.11, *p* = .735; Figure 1b). At the end of the experiment, nearly all the Clutch 1 gray treefrogs had metamorphosed, but most of the Clutch 2 gray treefrogs were still in the larval stage. Among the Clutch 1 gray treefrogs, there was a greater proportion of metamorphosing individuals captured at late stages (e.g. Gosner 43 and 44) in the early‐season treatment than in the late‐season treatment (*χ* ^2^ = 7.62, *p* = .006; Figure 1c). Clutch 1 gray treefrog metamorph total length (snout to tail tip) was significantly shorter in the early‐season (April) photoperiod while controlling for the development stage (photoperiod: *χ* ^2^ = 4.27, *p* = .039; stage: *χ* ^2^ = 228.95, *p* < .001; Figure 1d). Photoperiod did not affect Clutch 1 gray treefrog mass at metamorphosis (*χ* ^2^ = 0.22, *p* = .641), age at metamorphosis (*χ* ^2^ = 1.56, *p* = .211), snout‐vent length (*χ* ^2^ = 0.06, *p* = .805), head width (*χ* ^2^ = 1.75, *p* = .185), tibiofibula length (*χ* ^2^ = 0.02, *p* = .878), or total leg length (*χ* ^2^ = 0.17, *p* = .677). Photoperiod did not affect Clutch 2 gray treefrog larval mass (*χ* ^2^ = 1,10, *p* = .294), total length (*χ* ^2^ = 0.53, *p* = .467), or development stage (*χ* ^2^ = 1.62, *p* = .203). The median development stage for Clutch 2 gray treefrog larvae was Gosner 36 (Gosner, 1960).

### 3.3 Phytoplankton, periphyton, and zooplankton

For each sampling date, relative phytoplankton abundance measured as fluorescence did not vary between photoperiods (Day 10: *χ* ^2^ = 1.01, *p* = .315; Day 23: *χ* ^2^ = 0.20, *p* = .651; Day 36: *χ* ^2^ = 1.28, *p* = .257) or tadpole species (Day 10: *χ* ^2^ = 0.86, *p* = .353; Day 23: *χ* ^2^ = 0.01, *p* = .940; Day 36: *χ* ^2^ = 0.10, *p* = .751). Periphyton abundance measured as fluorescence from the tiles was significantly greater with gray treefrog tadpoles compared to green frog tadpoles (*F*_1,28_ = 5.99, *p* = .021). There was no effect of photoperiod treatment on periphyton (*F*_1,28_ = 0.15, *p* = .700).

**FIGURE 2:**
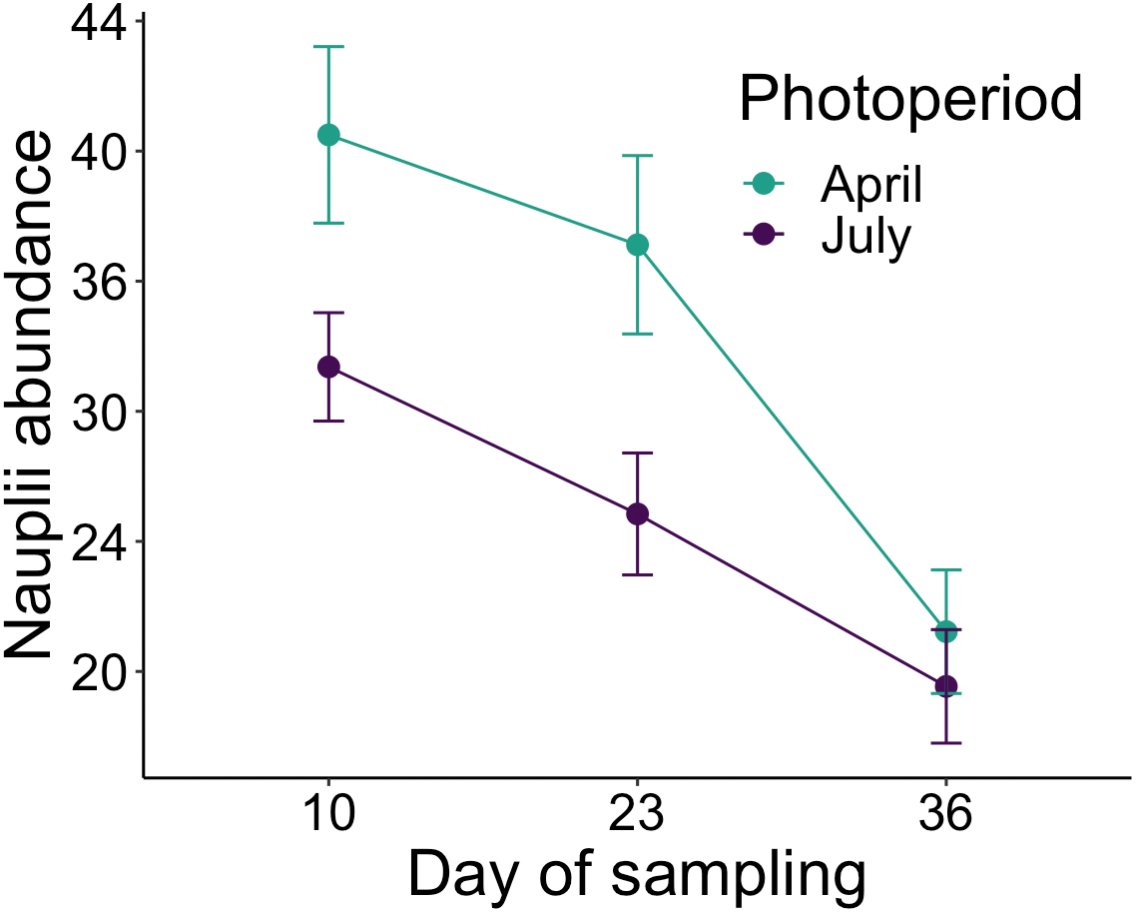
Copepod nauplii were in greater abundance under the early‐season (April) photoperiod on two of three sampling dates (Day 10: *χ* ^2^ = 5.06, *p* = .024; Day 23: *χ* ^2^ = 6.25, *p* = .012; Day 36: *χ* ^2^ = 0.15, *p* = .701). Means and standard error bars are plotted.

Photoperiod affected copepods in early life stages but no other zooplankton (Table A3). Copepod nauplii were in greater abundance under the early‐season (April) photoperiod on two of three sampling days (Day 10: *χ* ^2^ = 5.06, *p* = .024; Day 23: *χ* ^2^ = 6.25, *p* = .012; Day 36: *χ* ^2^ = 0.15, *p* = .701; Figure 2), regardless of frog species (Day 10: *χ* ^2^ = 0.10, *p* = .746; Day 23: *χ* ^2^ = 0.54, *p* = .463; Day 36: *χ* ^2^ = 0.28, *p* = .600). Photoperiod did not affect adult copepod abundance (Table A3); however, copepods were in greater abundance in gray treefrog mesocosms compared to green frog mesocosms on the final sampling day but not the other days (Day 36: *χ* ^2^ = 5.33, *p* = .021; Table A3). Neither cladocerans nor rotifers varied in abundance on any sampling day in response to photoperiod or species (Table A3). Additionally, zooplankton Shannon‐Weiner Diversity did not differ among photoperiods or species on any of the sampling days (Table A2).

## 4 DISCUSSION

As some organisms continue to shift phenology with rising temperatures, differences in how organisms respond to photoperiod may lead to changes in species interactions and community structure. We experimentally simulated a three‐month shift in photoperiod that affected both amphibian development and zooplankton abundance. In support of our prediction, green frogs accelerated development under the early‐season photoperiod. Although we had predicted that gray treefrogs would develop faster under the late‐season photoperiod, gray treefrogs instead accelerated development during metamorphosis under the early‐season photoperiod. Coinciding with increased development, gray treefrog metamorph total length decreased within development stages, indicating that gray treefrog metamorphs were more rapidly resorbing their tails under the early‐season photoperiod. Copepod nauplii also increased in abundance under the early‐season photoperiod. Despite these species‐specific effects of photoperiod, we did not find large shifts in species interactions nor the altering of community structure, suggesting that the freshwater organisms in our study that shift phenology are unlikely to respond to differences in photoperiod at this time interval (36 days). However, the effects of photoperiod may arise over longer time scales.

Photoperiod may be an important cue that drives development in larval amphibians, as green frogs were more developed under the early‐season photoperiod. This may represent an adaptive response to photoperiod such that green frogs use photoperiod as a seasonal cue to either metamorphose before the unfavorable season approaches or to overwinter as tadpoles (i.e. go/no‐go mechanism). Photoperiod provides go/no‐go signaling for promoting or ceasing diapause in many insects (Bradshaw & Holzapfel, 2007; Bradshaw & Lounibos, 1972). Alternatively, green frogs may have increased feeding under the shorter photoperiod treatment, as green frogs primarily forage at night (Fraker, 2008). Amphibians with greater food availability grow faster and metamorphose earlier (Alford & Harris, 1988; Wilbur & Collins, 1973). We did not detect effects of photoperiod on either mass or total length in green frog larvae, meaning that green frogs grew at the same rate. We therefore suspect that green frogs did not forage more under the early‐season photoperiod. Nonetheless, green frog larvae that metamorphose faster under early‐season conditions may have greater fitness in the post‐metamorphic terrestrial environment (Altwegg & Reyer, 2003).

Gray treefrogs accelerated metamorphic development under the early‐season photoperiod, which may be an adaptive response to minimize exposure to unfavorable thermal conditions or indicative of a fixed response to short photoperiods. We did not detect effects of photoperiod on the timing of the onset of metamorphosis (i.e. age at metamorphosis), meaning photoperiod solely affected gray treefrogs after initiating metamorphosis (presence of emerged front limbs). Metamorphosis is a sensitive life transition (Lowe et al., 2021); for example, amphibians have lower thermal tolerance during metamorphosis (Cupp, 1980; Enriquez‐Urzelai et al., 2019; Floyd, 1983). Gray treefrogs may have accelerated development during metamorphosis under early‐season photoperiod conditions to minimize exposure to elevated temperatures, but temperatures are greater in the summer than in spring, so this would seem unlikely. Alternatively, gray treefrog metamorphs may have accelerated development in response to shorter photoperiods, regardless of the directional change in photoperiod (increasing or decreasing). For example, daylengths in April are similar to that of August such that the total number of hours of light are similar, though they differ in directional change. Other organisms demonstrate changes in development resulting from differences in the total number of hours of light by comparing constant short versus long photoperiods (Johansson & Rowe, 1999; Kukita et al., 2015; Leimar, 1996; Śniegula & Johansson, 2010). Thus, the total number of hours of light in the shorter, early‐season photoperiod may have signaled late‐season conditions that cued gray treefrogs to metamorphose faster. This strategy would allow gray treefrogs to exit the pond faster during the late season when conditions are becoming less favorable.

Despite this impact of photoperiod on gray treefrog metamorph development, size and development of gray treefrog larvae (Clutch 2) did not vary in response to photoperiod. Gray treefrog larvae from the second clutch were added 8 days later than larvae from the first clutch and were exposed to a smaller difference in total light exposure within the photoperiod treatments, which may have been insufficient to induce differences in development rate. However, this seems improbable as there was approximately a two‐hour difference between the photoperiods when adding the second clutch, and other organisms have responded to smaller differences in photoperiod (Leimar, 1996; Śniegula & Johansson, 2010). We also preserved larvae from the second clutch during the late larval stage but not at metamorphosis. The lack of an effect of photoperiod on clutch 2 larval development further supports that photoperiod affected development only during metamorphosis in gray treefrogs. Alternatively, increased competition imposed on the second clutch by the first clutch (i.e. priority effect) may have masked the effects of photoperiod due to increased food restriction and growth limitation (Carter & Rudolf, 2019; Lawler & Morin, 1993; Murillo‐Rincón et al., 2017). Photoperiod did not affect growth rate in either gray treefrog clutches, so it is unlikely that resource restriction would have modulated the effects of photoperiod. Therefore, gray treefrogs likely responded to photoperiod only after initiating metamorphosis.

We did not detect strong effects of photoperiod on phytoplankton or periphyton, but copepod nauplii increased in abundance under the early‐season photoperiod. This suggests that photoperiod induced copepods to increase reproduction, though this increase did not persist into the third sampling date nor did it seem to affect other taxa in the community. The increase in nauplii abundance under the shorter photoperiod contrasts other experiments showing increased abundances under long photoperiods and relatively higher temperatures in copepods (Jones & Gilbert, 2016) and cladocerans (Dupuis & Hann, 2009; Jones & Gilbert, 2016). Our study did not manipulate temperature, and the water temperature in the mesocosms was much higher than the temperatures used in studies examining effects of photoperiod and temperature (Dupuis & Hann, 2009; Jones & Gilbert, 2016). Since photoperiod interacts with temperature (Dupuis & Hann, 2009; Jones & Gilbert, 2016), the results in our study may be specific to the temperature experienced in the mesocosms under these photoperiod conditions. We did not detect any effects of photoperiod on phytoplankton abundance, which differs from other experiments (Seyfabadi et al., 2011; Shatwell et al., 2012; Tang & Vincent, 2000; Theus et al., 2022). As expected, photoperiod did not affect periphyton abundance (Chaumet et al., 2020; Laderriere et al., 2022). However, we did find that periphyton abundance was higher at the end of the experiment in gray treefrog mesocosms, though this likely resulted from gray treefrogs having metamorphosed and ceased grazing upon the periphyton. Grazing by larvae has a strong effect on algal communities (Seale, 1980), so it is possible that the algal response to photoperiod was suppressed by larval grazing. However, we did not have a no‐larvae treatment to test this hypothesis. Additionally, because there are strong day/night cycles in larval feeding, it is possible that photoperiod treatments could have affected algal growth through larval feeding behavior (Fraker, 2008; Warkentin, 1992). Even though photoperiod had some effect on copepods, the remaining taxa in the community remained largely unchanged by the photoperiod manipulation.

Even though we detected developmental differences in two anurans and increased reproduction in certain zooplankton taxa, it is unclear to which aspect of photoperiod these organisms responded. We simulated two photoperiods that differ in direction of daylength change (increasing or decreasing) and the total number of hours of light exposure. It is possible these taxa responded to the change in daylength, the total number of hours of light in the day despite the incremental changes each day, or a combination of both properties. Other amphibians and zooplankton respond to constant long or short photoperiods (Dupuis & Hann, 2009; Jones & Gilbert, 2016; Kukita et al., 2015). A different temperate ranid than the one used in our study appears to not strongly respond to either aspect of photoperiod (Burraco et al., 2020; Laurila et al., 2001). Moreover, many fishes respond to constant photoperiods whereas others respond differently to photoperiods depending on previous photoperiod exposure, in terms of maturation and spawning (Bromage et al., 2001). Additionally, we did not alter light intensity to mimic the realistic light intensity (i.e. solar radiation) of the early‐season, as light intensity changes seasonally and is highest during summer (Sengupta et al., 2018). Few studies directly test for effects of light intensity on plankton abundance and their effects across freshwater communities (Dupuis & Hann, 2009). Seasonal changes in photoperiod, light intensity, and temperature may have important interactive effects on community dynamics, though the combined effects of these variables are rarely considered.

### 4.1 Conclusions

Although both amphibians and copepods responded to a photoperiod manipulation representing different seasons (spring and summer), the simplified freshwater community in our experiment did not exhibit significant changes to structure. Similarly, photoperiod disruption from artificial light at night had direct effects on amphibian development but did not alter interactions between amphibians and other members of the freshwater communities (Cope et al., 2020; Dananay & Benard, 2018). This is in strong contrast to how communities respond to other perturbations such as chemical contaminants (Miles et al., 2017; Rumschlag et al., 2020). However, our study shows that anurans from two different families with different reproductive strategies increased development under early‐season photoperiod conditions, suggesting that photoperiod may be an important seasonal cue driving development across temperate amphibians. As amphibians breed earlier in the year, offspring will develop under shorter, early‐season photoperiods which could result in faster development and thus have important implications for post‐metamorphic fitness. Photoperiod also affected zooplankton abundance in early life stages, which may affect species interactions throughout the food web over longer time scales. As organisms continue to shift phenology with rising temperatures, studies examining how different communities respond to variable photoperiod conditions, with other variables such as temperature and light intensity, will allow us to better project how these communities may shift under continuing global change.

## AUTHOR CONTRIBUTIONS

**Troy C. Neptune:** Conceptualization (equal); data curation (equal); formal analysis (lead); funding acquisition (equal); investigation (lead); methodology (equal); visualization (equal); writing – original draft (lead); writing – review and editing (lead). **Michael F. Benard:** Conceptualization (equal); data curation (equal); formal analysis (supporting); funding acquisition (equal); investigation (supporting); methodology (equal); visualization (equal); writing – original draft (supporting); writing – review and editing (supporting).

## FUNDING INFORMATION

This research was funded by the Case Western Reserve University (CWRU) Department of Biology Oglebay Fund.

## CONFLICT OF INTEREST STATEMENT

The authors declare that they have no conflicts of interest.

## Data Availability

Data used for analysis are available from the Dryad Digital Repository at https://doi.org/10.5061/dryad.vx0k6djxs.

## APPENDIX A

**TABLE A1:** Summary of photoperiods.

Date	Natural sunrise (AM)	Natural (late‐season) sunset (AM)	Simulated (early‐season) sunset (AM)	Natural (late‐season) daylength	Simulated (early‐season) daylength
30‐Jun	5:55	9:03	7:51	15:07:39	12:42:56
01‐Jul	5:55	9:02	7:52	15:07:01	12:45:41
02‐Jul	5:56	9:02	7:53	15:06:20	12:48:26
03‐Jul	5:56	9:02	7:54	15:05:34	12:51:10
04‐Jul	5:57	9:02	7:55	15:04:46	12:53:54
05‐Jul	5:58	9:02	7:56	15:03:53	12:56:38
06‐Jul	5:58	9:01	7:57	15:02:57	12:59:21
07‐Jul	5:59	9:01	7:58	15:01:58	13:02:04
08‐Jul	6:00	9:01	7:59	15:00:56	13:04:46
09‐Jul	6:00	9:00	8:00	14:59:50	13:07:28
10‐Jul	6:01	9:00	8:01	14:58:40	13:10:10
11‐Jul	6:02	8:59	8:03	14:57:28	13:12:50
12‐Jul	6:02	8:59	8:04	14:56:12	13:15:30
13‐Jul	6:03	8:58	8:05	14:54:54	13:18:10
14‐Jul	6:04	8:58	8:06	14:53:32	13:20:49
15‐Jul	6:05	8:57	8:07	14:52:07	13:23:27
16‐Jul	6:06	8:56	8:08	14:50:40	13:26:04
17‐Jul	6:06	8:56	8:09	14:49:09	13:28:41
18‐Jul	6:07	8:55	8:10	14:47:36	13:31:17
19‐Jul	6:08	8:54	8:11	14:46:00	13:33:52
20‐Jul	6:09	8:53	8:12	14:44:21	13:36:26
21‐Jul	6:10	8:53	8:13	14:42:40	13:38:59
22‐Jul	6:11	8:52	8:14	14:40:56	13:41:32
23‐Jul	6:12	8:51	8:16	14:39:10	13:44:03
24‐Jul	6:13	8:50	8:17	14:37:21	13:46:33
25‐Jul	6:14	8:49	8:18	14:35:30	13:49:02
26‐Jul	6:14	8:48	8:19	14:33:37	13:51:30
27‐Jul	6:15	8:47	8:20	14:31:42	13:53:57
28‐Jul	6:16	8:46	8:21	14:29:44	13:56:22
29‐Jul	6:17	8:45	8:22	14:27:45	13:58:47
30‐Jul	6:18	8:44	8:23	14:25:43	14:01:10
31‐Jul	6:19	8:43	8:24	14:23:40	14:03:31
01‐Aug	6:20	8:42	8:25	14:21:34	14:05:52
02‐Aug	6:21	8:41	8:26	14:19:27	14:08:10
03‐Aug	6:22	8:40	8:27	14:17:18	14:10:28
04‐Aug	6:23	8:38	8:28	14:15:07	14:12:43

*Note*: The natural (late‐season) photoperiod represents the unaltered daylength starting on 30 June 2021. The simulated (early‐season) photoperiod represents the daylength previously recorded each day starting on 1 April 2021. Data were obtained from timeanddate.com.

**TABLE A2:** Results of likelihood‐ratio tests testing for the effects of “photoperiod” and covariates on green frog (*Rana clamitans*) and gray treefrog (*Hyla versicolor*) traits in linear mixed models with Mesocosm as a random effect.

Outcome	Species	Clutch #	Predictor	*χ* ^2^	*p*‐Value	Estimate	SE	5% CI	95% CI
Development stage	*Rana clamitans*	N/A	Photoperiod	3.65	.056	−1.27	0.63	−2.3	−0.23
	*Hyla versicolor*	2	Photoperiod	1.62	.203	−1.56	1.21	−3.55	0.43
			Block	12.97	**.005**	–	–	–	–
Mass	*Rana clamitans*	N/A	Photoperiod	0.62	.431	0.02	0.02	−0.02	0.06
			Development Stage	512.61	**<.001**	0.1	<0.01	0.09	0.1
			Block	31.32	**<.001**	–	–	–	–
	*Hyla versicolor*	2	Photoperiod	1.10	.294	0.06	0.05	−0.04	0.15
			Development Stage	138.81	**<.001**	0.04	<0.01	0.03	0.04
Total length	*Rana clamitans*	N/A	Photoperiod	0.11	.735	0.21	0.63	−0.88	1.29
			Development Stage	426.28	**<.001**	2.05	0.07	1.93	2.17
			Block	21.23	**<.001**	–	–	–	–
	*Hyla versicolor*	2	Photoperiod	0.53	.467	0.66	0.9	−0.88	2.27
			Development Stage	249.14	**<.001**	1.81	0.07	1.69	1.93
Development stage	*Hyla versicolor*	1	Photoperiod	7.62	**.006**	−1.16	0.39	−1.81	−0.52
			Age at Metamorphosis	9.39	**.002**	0.33	0.11	0.15	0.52
Total length	*Hyla versicolor*	1	Photoperiod	4.27	**.039**	1.24	0.57	0.28	2.18
			Age at Metamorphosis	31.67	**<.001**	−1.19	0.18	−1.48	−0.88
			Development Stage	228.95	**<.001**	−10.03	0.48	−10.83	−9.24
Mass at metamorphosis	*Hyla versicolor*	1	Photoperiod	0.22	.641	−0.02	0.03	−0.07	0.04
			Age at Metamorphosis	23.25	**<.001**	−0.02	<0.01	−0.02	−0.01
			Development Stage	24.01	**<.001**	−0.04	0.01	−0.05	−0.03
			Block	10.43	**.015**	–	–	–	–
Age at metamorphosis	*Hyla versicolor*	1	Photoperiod	1.56	.211	0.61	0.48	−0.21	1.43
			Development Stage	9.93	**.002**	0.52	0.16	0.25	0.79
Snout‐vent length	*Hyla versicolor*	1	Photoperiod	0.06	.805	−0.06	0.24	−0.48	0.35
			Age at Metamorphosis	0.59	.444	−0.02	0.03	−0.07	0.03
			Development Stage	1.62	.204	0.09	0.07	−0.03	0.2
			Block	8.52	**.036**	–	–	–	–
Head width	*Hyla versicolor*	1	Photoperiod	1.75	.185	0.08	0.06	−0.02	0.19
			Age at Metamorphosis	1.51	.219	−0.02	0.01	−0.04	0.01
			Development Stage	14.97	**<.001**	−0.14	0.04	−0.2	−0.08
			Snout‐vent Length	49.23	**<.001**	0.22	0.03	0.17	0.27
Tibiofibula length	*Hyla versicolor*	1	Photoperiod	0.02	.878	0.01	0.07	−0.12	0.14
			Age at Metamorphosis	2.07	.150	0.02	0.01	<−0.01	0.04
			Development Stage	26.66	**<.001**	0.16	0.03	0.11	0.21
			Snout‐vent Length	83.97	**<.001**	0.25	0.03	0.2	0.31
			Block	10.19	**.017**	–	–	–	–
Total leg length	*Hyla versicolor*	1	Photoperiod	0.17	.677	0.12	0.29	−0.39	0.63
			Age at Metamorphosis	6.95	**.008**	0.11	0.04	0.04	0.18
			Development Stage	36.15	**<.001**	0.6	0.09	0.44	0.75
			Snout‐vent Length	104.06	**<.001**	0.91	0.1	0.74	1.08
			Block	10.78	**.013**	–	–	–	–

*Note*: Green frog and gray treefrog (Clutch 2) larval models are highlighted in green; gray treefrog metamorph (Clutch 1) models are highlighted in purple. There were no significant interactions. The estimate, standard error, and confidence intervals represent the July photoperiod compared to the April photoperiod. The estimate, standard error, and confidence intervals are not listed for each block.

Bolded *p*‐Values indicate values less than .05.

**TABLE A3:** Results of Likelihood‐Ratio Tests testing for the effects of “Photoperiod” and “Species” on zooplankton abundance and Shannon Diversity in linear mixed models with Mesocosm as a random effect, for each sampling day.

Outcome	Day of experiment	Predictor	*χ* ^2^	*p*‐Value	Estimate	SE	5% CI	95% CI
Cladoceran abundance	10	Photoperiod	0.04	.846	−0.03	0.13	−0.2	0.16
		Species	0.14	.713	−0.04	0.13	−0.22	0.14
		Block	10.87	**.012**	**–**	**–**	**–**	**–**
	23	Photoperiod	<0.01	.961	−0.01	0.17	−0.29	0.27
		Species	0.12	.727	−0.06	0.17	−0.34	0.22
		Block	9.18	**.027**	**–**	**–**	**–**	**–**
	36	Photoperiod	0.28	.594	0.08	0.15	−0.17	0.33
		Species	2.74	.098	−0.26	0.15	−0.51	−0.01
		Block	20.63	**<.001**	**–**	**–**	**–**	**–**
Copepod abundance	10	Photoperiod	0.14	.704	−0.04	0.1	−0.21	0.13
		Species	0.84	.360	0.09	0.1	−0.07	0.26
	23	Photoperiod	0.12	.729	0.04	0.12	−0.15	0.24
		Species	1.02	.313	−0.12	0.12	−0.32	0.07
	36	Photoperiod	0.02	.893	0.02	0.15	−0.22	0.26
		Species	5.33	**.021**	−0.36	0.15	−0.6	−0.11
Nauplii abundance	10	Photoperiod	5.06	**.024**	−0.22	0.09	−0.38	−0.07
		Species	0.10	.746	0.03	0.09	−0.12	0.19
	23	Photoperiod	6.25	**.012**	−0.33	0.13	−0.54	−0.12
		Species	0.54	.463	0.09	0.13	−0.12	0.3
	36	Photoperiod	0.15	.701	−0.06	0.16	−0.33	0.21
		Species	0.28	.600	0.09	0.16	−0.18	0.35
Rotifer abundance	10	Photoperiod	0.02	.902	−0.03	0.26	−0.45	0.39
		Species	0.14	.704	−0.1	0.26	−0.53	0.87
	23	Photoperiod	0.97	.324	0.42	0.43	−0.28	1.12
		Species	0.20	.657	0.19	0.42	−0.51	0.89
	36	Photoperiod	0.10	.756	0.17	0.54	−0.72	1.05
		Species	0.04	.850	−0.1	0.53	−0.98	0.78
Shannon‐Weiner diversity	10	Photoperiod	0.19	.661	−0.02	0.04	−0.08	0.05
		Species	1.09	.298	0.04	0.04	−0.1	0.02
	23	Photoperiod	2.04	.153	0.08	0.06	−0.01	0.17
		Species	1.67	.196	0.07	0.05	−0.16	0.02
	36	Photoperiod	0.36	.548	0.04	0.07	−0.07	0.16
		Species	0.02	.889	0.01	0.07	−0.12	0.11

*Note*: There were no significant interactions. The estimate, standard error, and confidence intervals represent the July photoperiod compared to the April photoperiod and the presence of green frogs compared to gray treefrogs. The estimate, standard error, and confidence intervals are not listed for each block.

Bolded *p*‐Values indicate values less than .05.
